# Neurophenomenological Investigation of Mindfulness Meditation “Cessation” Experiences Using EEG Network Analysis in an Intensively Sampled Adept Meditator

**DOI:** 10.1007/s10548-024-01052-4

**Published:** 2024-05-04

**Authors:** Remko van Lutterveld, Avijit Chowdhury, Daniel M. Ingram, Matthew D. Sacchet

**Affiliations:** 1 Brain Research and Innovation Centre and Department of Psychiatry, Ministry of Defence and University Medical Center, Utrecht, The Netherlands; 2 Center for Depression, Anxiety, and Stress Research, McLean Hospital, Harvard Medical School, Belmont, MA USA; 3 Emergent Phenomena Research Consortium, New Market, AL USA

**Keywords:** Cessation, Nirodha, Fruition, Mindfulness, Meditation, EEG

## Abstract

Mindfulness meditation is a contemplative practice that is informed by Buddhism. It has been proven effective for improving mental and physical health in clinical and non-clinical contexts. To date, mainstream dialogue and scientific research on mindfulness has focused primarily on short-term mindfulness training and applications of mindfulness for reducing stress. Understanding advanced mindfulness practice has important implications for mental health and general wellbeing. According to Theravada Buddhist meditation, a “cessation” event is a dramatic experience of profound clarity and equanimity that involves a complete discontinuation in experience, and is evidence of mastery of mindfulness meditation. Thirty-seven cessation events were captured in a single intensively sampled advanced meditator (over 6,000 h of retreat mindfulness meditation training) while recording electroencephalography (EEG) in 29 sessions between November 12, 2019 and March 11, 2020. Functional connectivity and network integration were assessed from 40 s prior to cessations to 40 s after cessations. From 21 s prior to cessations there was a linear decrease in large-scale functional interactions at the whole-brain level in the alpha band. In the 40 s following cessations these interactions linearly returned to prior levels. No modulation of network integration was observed. The decrease in whole-brain functional connectivity was underlain by frontal to left temporal and to more posterior decreases in connectivity, while the increase was underlain by wide-spread increases in connectivity. These results provide neuroscientific evidence of large-scale modulation of brain activity related to cessation events that provides a foundation for future studies of advanced meditation.

**Supplementary Information:**

The online version contains supplementary material available at 10.1007/s10548-024-01052-4.

## Introduction

Mindfulness meditation is a contemplative practice informed by Buddhism and involves non-judgmental, present-focused, moment-to-moment awareness of both one’s physical and mental states (Kabat-Zinn 2003). Over the past two decades, research has broadly supported the claim that mindfulness meditation exerts beneficial effects on physical and mental health, as well as cognitive performance (Grossman et al. 2004). Not surprisingly, therefore, the popularity of mindfulness meditation has burgeoned in recent years. Indeed, mindfulness is currently widely disseminated in clinical domains ranging across broad illness categories of physical and mental health (Salmon et al. 2004).

A branch of research on mindfulness meditation has tried to uncover the underlying neuroscience (Tang et al. 2015) by examining the associated brain areas and networks (Wheeler et al. 2017). Using electroencephalography (EEG), one of the most consistent findings to emerge from this line of studies is that mindfulness meditation is associated with an increase in alpha power relative to a resting state, along with a trend towards increased theta power (Fell et al. 2010; Lomas et al. 2015). Although alpha synchronization generally occurs during relaxed eyes-closed wakefulness and has widely been interpreted as the ‘idling’ of the brain, the combined theta and alpha synchronization associated with mindfulness meditation might reflect increased internalized attention – as theta synchronization is commonly viewed as a marker of cognitive control (Lomas et al. 2015). Studies using fMRI to examine mindfulness meditation have also revealed consistent activation in the right medial frontal gyrus/anterior cingulate and the left insula/inferior frontal gyrus (Falcone and Jerram 2018). Activation in these areas may reflect core components of mindfulness meditation including heightened self-regulation of attention and emotional processing (Tang et al. 2015).

Beyond these initial findings, there is a need for increasingly methodologically rigorous studies that will provide a more comprehensive understanding of meditation-induced changes in the brain. More specifically, meditation research should increasingly differentiate between the styles of meditation practice and the discrete stages of meditation within and between sessions, in addition to the proficiency of the meditator (i.e., novice vs. adept). Although many styles of meditation are often subsumed within the general rubric of “mindfulness meditation”, the two primary forms of mindfulness practice according to *Theravada* Buddhism are *Shamatha*/Concentration (focused attention) and *Vipassana*/Insight (open monitoring) (Lutz et al. 2007). These different forms of meditation implement unique methods for attention regulation and monitoring (Lutz et al. 2008), which are thought to be associated with distinct neural signatures. Thus, a better understanding of the style of meditation examined will facilitate an increasingly accurate interpretation of meditation research findings.

Additionally, different forms of meditation can be theoretically and experientially divided into specific states or stages that describe the sequential development of the sensate experience and cognition of the practitioner (Grabovac 2015). Indeed, generations of *Theravada* Buddhist practitioners and scholars have mapped experiences that emerge from regular meditation practice across thousands of years. Within *Vipassana* meditation, these experiences have been codified into discrete stages known as the Stages of Insight (Ñāṇamoli 2010). In this regard, one reason for inconsistent findings among different meditation studies is that they average measures from multiple stages of meditation together to determine mechanisms or effects or compare them without identifying the stage involved (Tang and Posner 2013). Finally, most meditation studies have examined participants with a wide range of experience with mindfulness, from entirely naïve to experts with decades of experience – which may further confound observed results.

In the present study, we used EEG to examine neural activity related to a unique state of the *Vipassana* meditation practice termed “cessation”as experienced by an extensively sampled adept meditator across multiple sessions. Cessations, or *nirodha* in Pali (the liturgical language of *Theravada* Budhism), are considered the culmination of the Stages of Insight and are described as the *phala* or fruit of meditation. Expert practitioners often report cessation as a momentary awareness of the extinction of experience. Following attainment of cessation, practitioners often note positive changes in perceptual abilities, psychological experience, and worldview (Grabovac 2015). In this context, cessations are an important research topic in understanding the beneficial effects of mindfulness meditation. Notably, cessation states tend to occur spontaneously during deep meditation after prolonged training and are relatively short episodes – typically lasting only several seconds (Sayadaw 1994). Two prior EEG studies have specifically examined the state of cessation: Berkovich-Ohana (2017) analyzed data from two adept meditators as they experienced three cessations each (i.e., a total of six cessations) and reported global long-range gamma (25–45 Hz) synchronization during states of cessation as compared to non-cessation states. Note that Berkovich-Ohana refer to these states as “fruition” events. In addition, Chowdhury et al. (2023) observed linear effects leading up to and following cessations in the alpha and theta frequency bands in a single adept meditator.

In our study, to obtain a sufficiently large number of cessation events and move beyond prior findings reported by Berkovich-Ohana (2017), we adopted a single intensive case design using a highly skilled and experienced meditator who could enter and report multiple cessation events as they emerge throughout repeated meditation sessions (Chowdhury et al. 2023). Using a neurophenomenological approach, systematic ‘first-person’ phenomenological criteria of cessations were used to select events for subsequent EEG-based analysis (Lutz and Thompson 2003). This approach of extensively sampling the event of interest (i.e., cessation) in a single participant has proved to be a powerful tool in neuroscience to derive insights beyond a single measurement (Poldrack et al. 2015). The participant for this study was ideal for this role, having extensive experience with the cessations and prior experience in neuroimaging environments (EEG, fMRI). Here we used a functional connectivity and graph theory approach to assess the brain’s network integration within time windows before and after cessations. Based on our prior studies (van Lutterveld et al. 2017) and other studies that have identified meditative states to be associated with alpha power (Fell et al. 2010; Lomas et al. 2015), we hypothesized that cessation events would be associated with the large-scale modulation of brain activity in the alpha band.

## Materials and Methods

### Participant

The participant was co-author DMI: a male meditation teacher and adept with 26 years of meditation experience at the time of data acquisition, and an estimated over 6,000 h of time spent in meditation retreats. He was 50–51 years old at the time of data collection. The study was performed in line with the principles of the Declaration of Helsinki and was approved by the Western Institutional Review Board (WIRB), and the participant provided informed consent.

### EEG

#### Data Acquisition

**Fig. 1:**
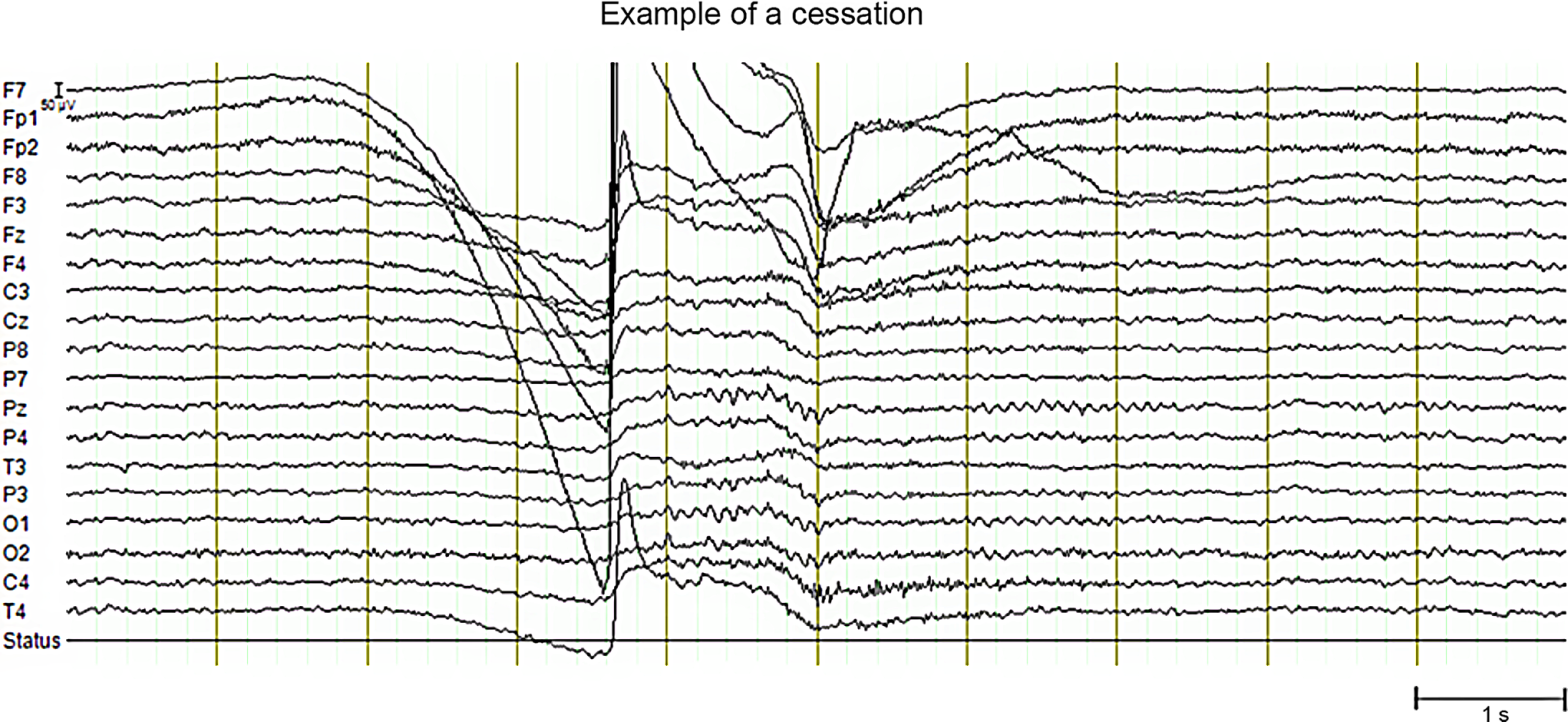
Example of a cessation with accompanying artifact. Artifactual data surrounding the cessation were omitted from analysis

EEG data were recorded with a Quick-20 EEG system using a cap with 19 dry electrodes (Cognionics, San Diego, CA, USA) using a sampling rate of 500 Hz. Twenty-nine EEG runs were acquired (length ranging between 426 and 3,653 s) while the participant performed meditation of two primary types (*Vipassana*, specifically “review practice”, where the inclination was to the target of cessation, and *fire kasina*, a traditional *Theravada* practice focusing on internally-generated visual sensations) with eyes closed. For sixteen runs, Vipassana meditation was performed, and for thirteen runs, fire kasina meditation was performed. Cessations were accompanied by an involuntary movement artifact which also contained a large eye blink that can be easily distinguished from the rest of the run that is conducted with eyes closed. We are unaware of the etiology of these involuntary eye-blinks coinciding with cessations. Anecdotal reporting from other advanced meditators suggests that the subject’s response to cessations may be idiosyncratic and that in other meditators cessations may be accompanied by a combination of eye movements and body twitches (Chowdhury et al. 2023). We also ensured that our identification of the cessation was accurate by examining the inter-rater reliability in marking the time of the eye-blink: the participant and one of the authors independently inspected the EEG runs to assess the onsets of cessations and reported the same location for the eye-blinks. This eye-blink approach was validated in a run with seven cessations in which cessations were marked in the time frame afterwards by button-press. Figure 1 provides an example of a cessation.

Button-presses were excluded from the other runs, which minimized confounding monitoring processes (Van Lutterveld et al. 2013). Immediately after each run, the quality of the cessations was graded by the participant. Here we employed a neurophenomenological approach in the broad sense, where systematic ‘first-person’ descriptions of experience are related to objective or ‘third-person’ neuroimaging data (Lutz and Thompson 2003). Specifically, our phenomenologically trained subject systematically evaluated the mental and physiological processes relevant to cessations as he experienced them, and these evaluations were used to classify and select events (i.e., high grade cessations) for subsequent EEG-based analysis. Criteria for assessment were as follows: Grade A cessations included setup, entrance, the cessation itself, exit, and afterglow, with each of these factors clear and correct. Grade B was characterized by the same factors as grade A, but at least one factor was not perfectly clear. Grade C cessations were associated with unclear factors. See supplementary text S1 for additional information on the description and grading of cessations. Forty-six Grade A, twelve Grade B, and eleven Grade C cessations were acquired. The EEG runs contained a varying amount of cessations (average total number of cessations per run: 2.38 [range 1–8], average number of Grade A cessations: 1.59 [range 0–8], average number of Grade B cessations: 0.41 [range 0–3], and average number of Grade C cessations: 0.38 [range 0–3]). Supplementary Table S2 provides an overview of the distribution of the cessations across runs and meditation types. We selected grade A cessations for further analysis as these most likely represented the purest form of cessations. For the same reason, we did not compare EEG activity between the different grades of cessation as there would be considerable heterogeneity in how Grade B or C cessations differ from Grade A cessations. In addition, Grade A cessations were the most ubiquitous of the three grades and as such presented the most statistical power. To minimize carry-over effects from adjacent cessations, additional criteria included an interval of at least at least 80 s from other cessations. Furthermore, cessations were required to occur at least 40 s after the start or 40 s before the end of the run. One dataset was excluded from analysis because of technical difficulties. Subsequent analyses were thus conducted on 37 cessations that met inclusion criteria. For the control analysis, control markers were added to the runs. These markers were required to meet the following criteria: being at a position of at least 40 s after the start or 40 s before the end of the run, being at a position of at least 160 s from the nearest cessations to minimize chances of carry-over effects from cessations. All time-segments that were long enough to meet these criteria were identified, and for each of these time-segments, a control marker was added at a random position, with a total of 27.

#### Preprocessing

Data were analyzed in a manner similar to van Lutterveld et al. (2017). Briefly, the BrainVision Analyzer software suite was used for preprocessing (BrainProducts, Munich, Germany). Data were visually inspected for bad channels, which were subsequently removed from analysis (see supplementary text S3). EEG data were filtered using a Butterworth Infinite Impulse Response (IIR) filter between 0.5 and 100 Hz with a slope of 48 dB/octave, and subsequently segmented in segments of 1024 samples (2.048 s) in the 39.936 s pre-cessation to 39.934 s post-cessation timeframe. After this, automated artifact identification was performed using the following parameters: (1) maximal allowed voltage step: 50 µV/ms; (2) maximal allowed difference of values in 200 ms intervals: 200 µV; (3) minimal allowed amplitude: −100 µV and maximal allowed amplitude: 100 µV; (4) lowest allowed activity in 100 ms intervals: 0.5 µV; All data were visually inspected by an experienced EEG scientist (RvL) to identify artifacts in the data that were not picked up by the automated artifact rejection algorithm, these were mainly motion/muscle activity. Segments containing artifacts were removed from analysis.

#### Functional Connectivity

BrainWave software (version 0.9.152.12.26) was used for functional connectivity and graph analysis (http://home.kpn.nl/stam7883/). Data were band-pass filtered in the delta (1.5–4 Hz), theta (4–8 Hz), alpha (8–13 Hz), and beta (13–20 Hz) frequency bands using fast Fourier transform (FFT) to produce a so-called zero-phase shift “brickwall” filter. Frequencies above 20 Hz were not included in the analysis because of contamination with muscle activity (Whitham et al. 2007; Yuval-Greenberg et al. 2008). Data were re-referenced to average reference. Functional connectivity between all scalp electrodes was calculated using the phase-lag index (PLI; Stam et al. 2007a). The PLI measures phase synchronization between time series by assessing the nonzero phase-lag between those time series, and ranges between 0 (no phase synchrony) and 1 (complete phase synchrony). Importantly, the PLI has specifically been developed for EEG analysis and is less likely to be confounded by volume conduction (Porz et al. 2014; Stam et al. 2007). It has been used extensively to study functional connectivity and graph analysis using EEG and magnetoencephalography (MEG)(e.g. López et al. (2017), He et al. (2019), Tóth et al. (2017), Van Montfort et al. (2020), including in meditation research (van Lutterveld et al. 2017). In the present study, overall functional connectivity was calculated by averaging between all pairwise PLI measurements. As at least six to ten 2-second segments are required to establish a stable phase lag index (PLI) signal in EEG meditation research (van Lutterveld et al. 2017), we only included segments for which at least ten segments for that timepoint survived artifact rejection. For this reason, the segment containing the cessation as well as the segments directly preceding and following the cessation were omitted from analysis.

#### Network Integration

The PLI network was assessed using graph theory. To assess network integration, diameter of the minimum spanning tree (MST) was calculated in BrainWave. The MST is the binarized subgraph of the weighted connectivity matrix, containing all nodes and the strongest associations, without forming loops (Stam et al. 2014). Essentially, the MST is a structure that can be considered the functional backbone of the original network. Importantly, the MST provides a relatively unbiased comparison between networks (Van Wijk et al. 2010; Tewarie et al. 2015). Diameter of the MST represents the length of the longest path in the tree. It is negatively associated with network integration, with a low diameter signifying high network integration and efficient information flow (Stam et al. 2014; Tewarie et al. 2015; van Dellen et al. 2016). Diameter values were normalized for network size, ranging from 0 to 1.

#### Statistical Analysis

**Fig. 2:**
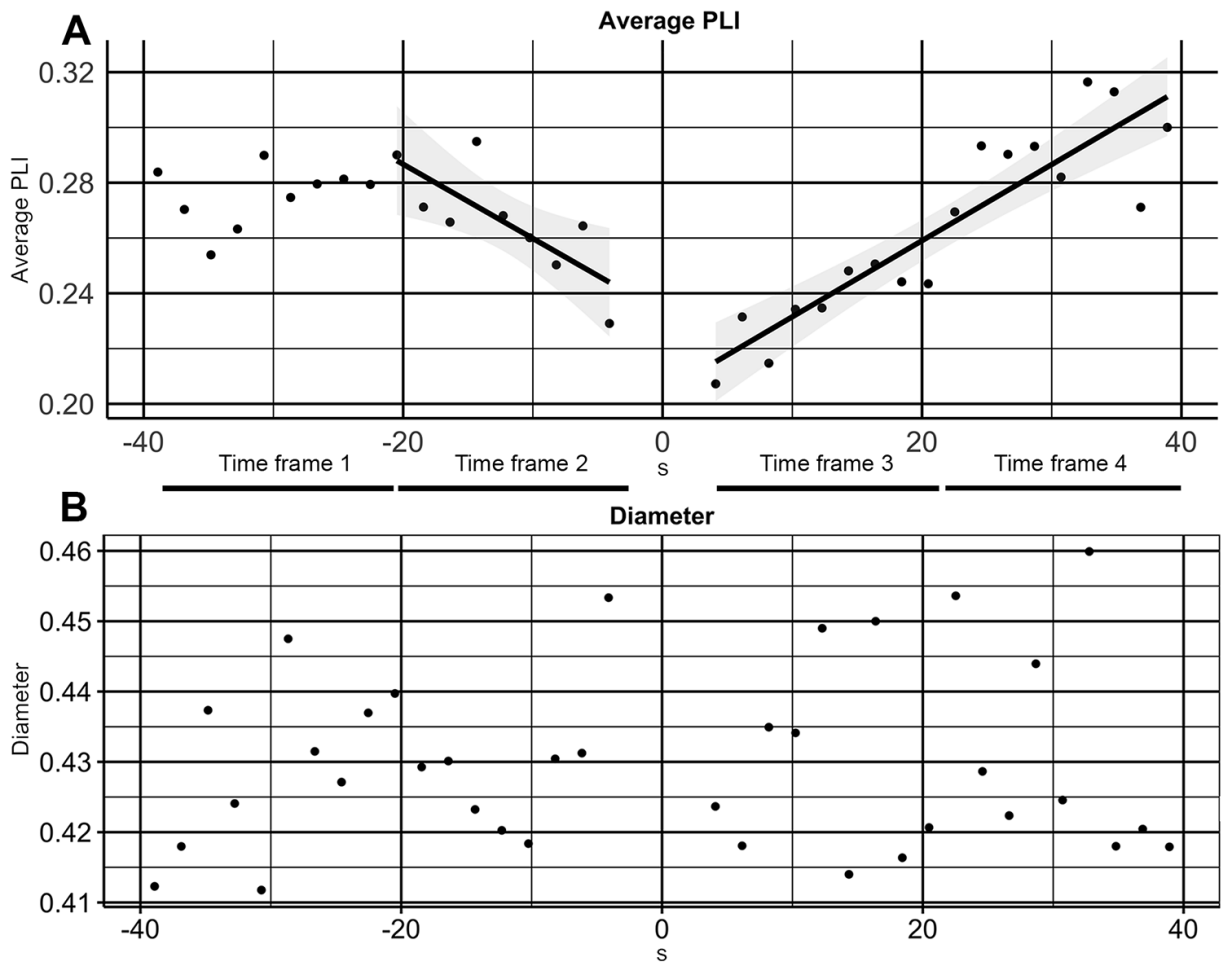
Functional connectivity and network integration in the alpha band for the cessation analysis. The 0 s timepoint indicates when the cessation occurred. The lines indicate the significant regressions and the grey shading indicates 95% confidence intervals. PLI: Phase-lag index

All statistical analyses were performed using SPSS version 25.0.0.1 (IBM SPSS Inc., Chicago, IL, USA). First, data were binned in four consecutive time frames. Time frame 1 ran from 39.936 s to 21.506 s pre-cessation and time frame 2 from 21.504 to 3.074 s pre-cessation while time frame 3 ran from 3.072 to 21.502 s post-cessation and time frame 4 from 21.504 to 39.934 s post-cessation. For the control analysis, binning in time frames was performed relative to the control marker in an identical fashion. The linear bars at the bottom of Fig. 2A provide a graphical representation about the timing of the time frames.

As previous research found several markers of network integration (including the network measure diameter) in the alpha band to be associated with meditation (van Lutterveld et al. 2017), modulation of functional connectivity and network integration in the alpha frequency band was analyzed in several ways: (1) To assess differences across time frames, overall PLI and diameter values were assessed over the four time frames using Friedman’s test, with *post hoc* testing using Wilcoxon signed-rank tests with correction for multiple comparisons using False Discovery Rate (FDR, Benjamini and Hochberg 1995). (2) If step 1 revealed modulation, linear regressions were performed to explore linear changes, with time as a predictor and overall PLI or diameter as the dependent variable. Separate regression analyses (with 2000 bootstraps) were performed for (2a) the entire timeframe leading up to cessations and the timeframe after cessations (containing either both time frame 1 and 2, or both time frame 3 and 4). (2b) If no predictive value of time was observed pre-cessation or post-cessation, the analysis was repeated using the time frame immediately leading up to the cessation or after the cessation (i.e., time frame 2 or time frame 3). Regression results were corrected for multiple comparisons using FDR correction. In addition, all analyses were repeated for the exploratory analyses in the delta, theta, and beta frequency bands.

Significant whole-brain regression results were further explored by conducting regression analyses for the Vipassana runs and fire kasina runs separately, and by repeating the regression analysis for average functional connectivity (PLI) values between 7 regions of interest (ROIs) for time frames in which significant regression overall PLI findings were observed. These ROIs encompassed electrodes in the frontopolar (Fp1, Fp2), frontal (F3, F4, Fz, F7, F8), central (C3, Cz, C4), parietal (P3, Pz, P4), left temporal (T3, T5), right temporal (T4, T6) and occipital (O1, O2) regions (see Supplementary figure S4 for a graphical representation). Average PLI between ROIs was calculated by averaging all possible pairwise PLI values from electrodes within one ROI to another. Because of the exploratory nature of this analysis, no correction for multiple comparisons was performed. For the control analyses, Friedman’s test was performed identical to the cessation analysis, and overall PLI and ROI linear regressions were performed identically for the significant time frames in the cessation analyses. Visual representations of the data were created using the R ggplot2 package (Wickham 2009).

## Results

For the cessation analysis, Friedman’s test revealed a significant difference in overall PLI in the alpha band across the four time frames (*χ*^2^(3) = 14.467, *P* = 0.002). Post-hoc testing showed a significant difference between time frames 3 and 1 (*Z* = -2.666, *P*_corrected_ = 0.024), and time frames 3 and 4 (*Z* = -2.666, *P*_corrected_ = 0.024). No significant differences were observed between time frames 1 and 2 (*Z* = -1.007, *P*_corrected_ = 0.314), time frames 1 and 4 (*Z* = -1.955, *P*_corrected_ = 0.061), time frames 2 and 3 (*Z* = -2.192, *P*_corrected_ = 0.056), and time frames 2 and 4 (*Z* = -2.073, *P*_corrected_ = 0.057). No significant effects were observed for the control analysis (*χ*^2^(3) = 5.000, *P* = 0.172). Linear regression revealed that time did not significantly predict overall PLI in the entire pre-cessation time frame (time frames 1 and 2 combined), while it did in the time frame immediately preceding cessation (time frame 2). Time also significantly predicted overall PLI in the entire post-cessation time frame (time frames 3 and 4 combined). Overall, these findings show that overall PLI is reduced in the time frame directly following the cessation compared to its subsequent time frame as well as the first time frame. Moreover, overall PLI linearly decreases from 21.504 to 3.074 s pre-cessation and linearly increases from 3.072 to 39.934 s post-cessation. No linear effects within these time frames were observed for the control data (*P*-values 0.760 and 0.842). Figure 2 provides a graphical representation of the PLI and diameter findings in the alpha frequency band, and Table 1 provides model statistics for the regression analysis. while supplementary figure and text S5 provide a graphical representation and model statistics for the Vipassana and fire kasina runs separately. Friedman’s test revealed no significant differences for diameter (*χ*^2^(3) = 0.333, *P* = 0.954).

**Table 1:** Model statistics for the overall phase-lag index (PLI) regression analyses in the alpha band for the cessation analysis. CI: confidence interval. The model statistics *P*_*corrected*_ column represents *p*-values corrected for multiple comparisons

	Model statistics				Parameter estimates			
Time frames	Degrees of freedom	*F*	*R* ^2^	*P*_*corrected*_	B(constant) (BCa 95% CI)	*p*	B(time) (BCa 95% CI)	*p*
Pre-cessation (39.936–3.074 s pre-cessation; time frames 1 and 2 combined)	1,16	3.505	0.180	0.080	0.257 (0.239–0.282)	< 0.001	-0.001(-0.001–0.000)	0.140
Pre-cessation (21.504–3.074 s pre-cessation, time frame 2)	1,7	9.951	0.587	0.024	0.233 (0.210–0.264)	0.002	-0.003 (-0.005–0.000)	0.039
Post-cessation (3.072–39.934 s post-cessation; time frames 3 and 4 combined)	1,16	70.115	0.814	< 0.003	0.204 (0.191–0.216)	< 0.001	0.003 (0.002–0.004)	0.001

### ROI Analyses

**Fig. 3:**
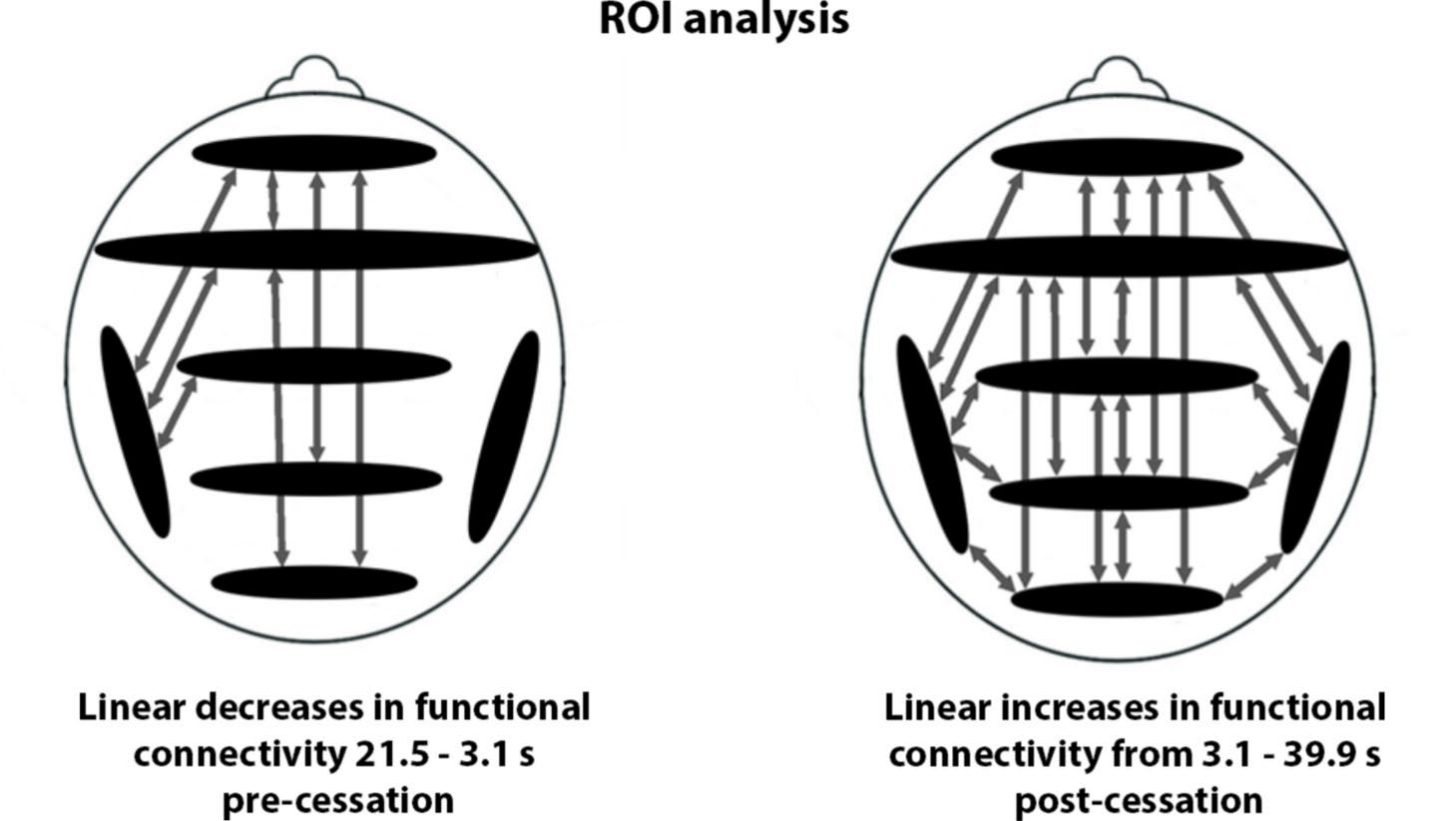
Results of the functional connectivity region-of-interest (ROI) analysis. The arrows in the left panel indicate significant linear decreases in functional connectivity from 21.5 to 3.1 s pre-cessation, while the arrows in the right panel indicate significant linear increases in functional connectivity from 3.1 to 39.9 s post-cessation. No significant findings were observed in the control data

The exploratory ROI analysis revealed linear decreases between 7 ROI-pairs (out of a total of 21 ROI-pairs) from 21.504 to 3.074 s pre-cessation, primarily from frontal areas to the left temporal lobe and to more posterior regions. In addition, linear increases between 20 ROI-pairs (out of a total of 21 ROI-pairs) from 3.074 to 39.934 s post-cessation were observed. No linear relationships between ROIs within these time frames were observed for the control data. Figure 3 provides a graphical representation of the ROI results, and Supplementary Table S6 provides p-values per ROI-pair.

### Other Analyses

For the exploratory cessation analyses investigating overall PLI and diameter in the delta, theta, and beta frequency bands, Friedman’s tests revealed no significant differences (*P*-values ranging from 0.435 to 0.769). For the control data, no significant differences in overall PLI were observed across the four time frames for these frequency bands (*P*-values ranging from 0.228 to 0.769). Friedman’s test also revealed no significant differences in diameter across the four time frames in any frequency band for the control data (*p*-values ranging from 0.435 to 0.896).

These results show that cessations are preceded by a linear decrease in overall functional connectivity in the alpha band from 21.504 to 3.074 s pre-cessation, which is associated with a linear decrease in frontal to left temporal and to more posterior regions. These results also show that cessations are followed by a linear increase in overall functional connectivity in the same band from 3.074 s to 39.934 s post-cessation, which is associated with widespread linear increases in functional connectivity across the brain.

## Discussion

This is the one of the first studies to examine the neuroscience of extensively sampled cessations as they occurred during mindfulness meditation. We found that from 21 s prior to cessation, a linear decrease starts in large-scale functional neural interactions as reflected by an EEG-based PLI. Markedly, this interaction was lowest immediately following a cessation. After cessation, these interactions returned to prior levels. The modulation of network integration was unique to the alpha frequency band – we did not observe any differences in functional neural interactions before or after cessations in the delta, theta, or beta frequency bands. By relating cessation events to large-scale modulation of brain activity, these results provide validity and lay the foundation for studying individual states of mindfulness meditation from a neuroscientific approach. Furthermore, our study informs future research of advanced meditation and ultimately promises to inform training programs that will facilitate therapeutic outcomes in both clinical and non-clinical populations.

The current results support our hypothesis of large-scale modulation of brain activity in the alpha band surrounding cessations by showing a linear decrease in network connectivity in the alpha band 21 s before the cessation event. This decrease may indicate a gradual reduction in information exchange between different brain areas and ultimately to the experience of a ‘cut’ from consciousness during cessations. Notably, in van Lutterveld et al. (2017) we previously reported increased alpha-band network integration during meditation in experienced meditators. Our results suggest that although, on average, a highly adept meditator may exhibit greater brain network integration during meditation, cessation events are experienced following a gradual decrease in overall brain connectivity. In general, these findings highlight the need for examining distinct stages/states of meditation, as it can help to relate the state-specific phenomenological experience to its underlying neural representation. For example, as we focused on an advanced state of meditation in the *Theravada* practice, future studies may compare similar states from other meditation traditions. This would provide a better understanding of how theoretical, phenomenological, and neural characteristics of these states converge across traditions.

These findings are partially in line with spectral analysis of the time frame surrounding cessations (Chowdhury et al. 2023). Using the same data and a similar statistical analysis, a linear decrease in the 39.936–3.074 s time frame leading up to cessations in alpha-band power was observed, as well as a linear increase in the 3.072–39.934 s time frame following cessations. Further region-of-interest (ROI) examination of these findings revealed that this specific pattern of alpha-suppression was observed in the occipital and parietal regions of the brain. In addition, linear increases following cessations were observed in the frontopolar, central, and left and right temporal areas, while no linear decreases were observed preceding cessations in these regions. Interestingly, this study also implicated the theta band. A linear increase in whole-brain power in the 39.936–3.074 s preceding cessations was observed, while no linear effect was observed after cessations. This effect was carried by central, parietal, and right temporal ROIs.

Interestingly, the phenomenology of advanced meditation states such as cessations overlaps with that of psychedelic experiences (Millière et al. 2018). Both are altered states of consciousness commonly associated with a reduced sense of self (Nour et al. 2016; Berkovich-Ohana 2017). This similarity in subjective experience is paralleled by comparable findings on the effects of psychedelics (e.g., Psilocybin, LSD) on the brain – which is a diminished hierarchical organization of brain networks induced by greater crosstalk between low-level sensorimotor and abstract cognition networks (Girn et al. 2021). Psychedelics may bring about this decrease in modular network activity through lowering of region-specific synchronizations in the brain (Riba et al. 2004; Muthukumaraswamy et al. 2013). For example, Psilocybin has been shown to decrease activity in the default-mode network and may be an effective treatment for depression (Daws et al., Under review). Future research should examine whether the decrease in synchronization in our study during cessations is similar in nature and effect to psychedelics, for example, whether the effects are regional or global, and how it may relate to clinical features (e.g., symptoms of depression). In relation to their potential therapeutic use, both psychedelics and advanced meditative states may reduce attention allocated towards pathologically overweighted prior experiences (Carhart-Harris and Friston 2019; Laukkonen and Slagter 2021) – thus enabling revision of beliefs and generation of novel insights.

To the best of our knowledge, to date, only one previous EEG study (Berkovich-Ohana 2017) has investigated the neuroscience of cessations using a different data set. That study reported a significant increase in overall long-range gamma (25–45 Hz) synchronization with a sample of only six cessation events. Differences between this finding and the observation of decreased synchronization in the alpha band in the present study may be explained by the current study’s significantly greater number of cessation events, the difference in signaling method used by the meditators to indicate a cessation event (i.e., moving eyes from left to right), the use of different meditators in their study which might render the experience of cessation differently, and differences in computational methods including the applied network integration measures. Notably, gamma synchronization in humans is similar in terms of its frequency characteristics with electrical muscle activity recorded by EEG (Sauseng and Klimesch 2008), and thus the results from Berkovich-Ohana (2017) may be confounded with non-brain physiological signals, particularly due to that study’s small number of cessations. Overall, in the current study, we limit our interpretation away from gamma to avoid interpreting possible muscle artifacts.

### Limitations

The current study used a single case design in which we extensively sampled cessation events from a single adept meditator (cessation *N* = 37). Despite this design being a major strength of the study, such a protocol simultaneously reduces the generalizability of the current results. Future studies with multiple participants of varying levels of meditation practice will allow for the examination of the broader validity of the current findings. It is also important to note that cessations were accompanied by a relatively strong motion artifact (i.e., eye-blink). To the best of our knowledge, the effect of motion correction algorithms such as independent component analysis (ICA) on the phase-lag index has not yet been established. Thus, we conservatively excluded these segments from the current analysis. Future studies that control for such motion artifacts will be instrumental for advancing our understanding of the neural signals at the precise moment of cessation. The duration of a cessation, by having the participant indicate the end of a cessation, was also not measured in this study. The eye-blink at the start of a cessation is involuntary, whereas another indicator to mark the end of cessation would have to be voluntary and therefore burden the participant with additional monitoring requirement, making it difficult to experience a cessation. The small number of EEG channels in the present study deters us from inferring on the specific locations of brain activity/connectivity and we report whole brain analyses here. However, we contend that the present findings are uniquely informative since large-scale PLI-based connectivity measures have previously been associated with meditation (van Lutterveld et al. 2017).

## Conclusions

In sum, these results provide neuroscientific evidence for the large-scale modulation of brain activity related to cessation events. We provide groundwork for future studies that aim to examine distinct altered states of consciousness that emerge from advanced meditation and related conditions (e.g., psychedelic states), and ultimately translate into more effective meditation training programs for both clinical and non-clinical populations.

## Electronic Supplementary Material

Below is the link to the electronic supplementary material.

Supplementary Material 1

## Data Availability

The original data is available in a limited open source model and will be made available by the authors in accordance with the guidelines approved by the Western Institutional Review Board (WIRB). Requests to access the datasets should be directed to msacchet@mclean.harvard.edu.
