# Supplementary material for: Neurophenomenological Investigation of Mindfulness Meditation “Cessation” Experiences Using EEG Network Analysis in an Intensively Sampled Adept Meditator
Source: Brain Topogr. 2024 May 4;37(5):849–58. doi: 10.1007/s10548-024-01052-4 (PMC11393101; doi:10.1007/s10548-024-01052-4)
Supplement: Supplementary file 1 — Supplementary Material 1 [file 10548_2024_1052_MOESM1_ESM.docx]

Supplementary text S1

*Phenomenology and Cessation Grading Criteria*

Cessations were identified, differentiated from other experiences, and graded using the following five cessation
components and associated criteria. Major criteria of a given component were required to meet a given grade.
Minor criteria of a given component supported the classification of major criteria but were not necessary to
satisfy meeting criteria for a given component.

1. Setup:
*Major:*
1. Clear progression through the entire Stages of Insight (Ñāṇamoli, 2010).
*Minor:*
1. Experience of formlessness within minutes prior to the Cessation.
2. Awareness of all of experience beginning to synchronize in “gently rocking” manner.
3. Awareness of entering into “3D” visual experiences or other visual phenomena that tend to precede
Cessations, such as vivid 3D moving landscapes or 3D rotating objects.

2. Entrance:
*Major:*
1. Experiential clarity of Three Door phenomenology. This major criterion includes six basic presentations that are combinations of the three elements of: (1) the collapse of the sense of object and subject (i.e., no-self), (2) the sense of the complete and somewhat jarring separation of experience from the sense of awareness (suffering), and (3) the complete pulsing of all experience (impermanence). A simplified explanation of the six presentations is: (1) the No-Self/Impermanence Door is the collapse of subject and object and is perceived in 3-4 complete pulses of all of experience (2) The Suffering/Impermanence Door is the removal of experience from awareness and is perceived in 3-4 pulses of all of experience, and (3) the No-Self/Suffering Door, in which experience rotates, subject
and object appear to trade places, and experience vanishes when this is complete. Each of these
doors may be reversed if one or the other characteristic is more predominant, yielding the
Impermanence/No-self Door, the Impermanence/Suffering Door, and the Suffering/No-Self Door.
There are other more rare phenomenological variants of Three Door phenomenology and they follow
similar themes, and all appear to have the same outcome. These Doors tend to take approximately
100 to 300 ms as estimated experientially. Each Door involves the phenomenological sense of
involving all of experience, with the sense of complete, totally inclusive, undivided, and synchronized
attention that is called Conformity Knowledge (Ingram, 2018).
2. Occurance of fluttering eyelids.

3. Event:
*Major:*
Experience entirely gone for the cessation event. A clean sense of the discontinuity of experience
based on the memories of the sensations before and after cessation and their disjoined relationship.

4. Exit:
*Major:*
1. Mind experienced as “freshly restarting”, having been “deeply reset”. Both body and mind feeling
refreshed, satisfying completion, and clean in a way that only happens after cessations.
2. Pattern of breathing changed in the predictable way such that there was a sense of breathing starting
on the inbreath and moving in a deeper, more fluid way immediately afterwards.
3. Experience did not have “analogue power-down then power-up” characteristic of Nirodha Samapatti,
which involves the rapid, smooth (non-strobing, thus analogue (rather than discrete/digital)), complete
cessation of consciousness of internal verbal formations, bodily sensations, and then feeling and
perception itself (Ñāṇamoli, 2010), with the exit occurring in the reverse order and taking
approximately the same amount of time.

5. Afterglow/Aftereffects:
*Major:*
1. Experience of “afterglow” over seconds to minutes of increasing rapture, refreshment, and mental
cleanliness.
2. Afterglow was not like the deeper, stronger, and longer-lasting afterglow of Nirodha Samapatti.
*Minor:*
1. Experience of visuals routinely noticed immediately after cessations in this particular practitioner (e.g. a
lighter purple circle on a darker purple background).
2. Mind re-inclined toward additional cessations.
3. Mind returned to the beginning of a cycle of the Stages of Insight.

*Grades:*
A. If all five components (and all major criteria therein) were met with a high degree of certainty, the given cessation was classified as Grade A.
B. If four of the five components were met with a high degree of certainty, but one component was met with a moderate degree of certainty, the given cessation was classified as Grade B. Often, Grade B cessations did not meet Grade A classification because they did not include perception of the Entrance with a high degree of certainty that it met the phenomenology of the Three Doors.
C. If there is still belief that a cessation may have occurred, but the phenomenology was not clear enough to meet criteria for Grades A or B, then the given experience was classified as Grace C. This may occur
when the Exit and Afterglow/After-effects were clear, but the Entrance was not, in addition to other
features not having been clear. For example, uncertainty regarding the completeness of the experiential
discontinuity (The Event), the Event occurred early in the Setup without clear final Stages of Insight (e.g., Equanimity), or the Entrance Door was not clearly perceived.
D. If the Exit and Afterglow criteria were not met, the given experience was not considered for further
evaluation and was thus not graded as a cessation.

Supplementary table S2. Distribution of cessations across runs and meditation types.

| Run number | Meditation type | Nr of Grade A cessations | Nr of Grade B cessations | Nr of Grade C cessations |
| --- | --- | --- | --- | --- |
| 1 | Vipassana | 1 | 0 | 3 |
| 2 | Vipassana | 1 | 0 | 0 |
| 3 | Vipassana | 1 | 0 | 0 |
| 4 | Vipassana | 1 | 0 | 0 |
| 5 | Vipassana | 1 | 0 | 0 |
| 6 | Vipassana | 1 | 0 | 0 |
| 7 | Vipassana | 0 | 1 | 0 |
| 8 | Vipassana | 1 | 0 | 0 |
| 9 | Vipassana | 1 | 0 | 0 |
| 10 | Vipassana | 1 | 0 | 0 |
| 11 | Vipassana | 1 | 0 | 0 |
| 12 | Vipassana | 1 | 0 | 1 |
| 13 | Vipassana | 1 | 0 | 0 |
| 14 | Vipassana | 1 | 0 | 0 |
| 15 | Vipassana | 1 | 1 | 0 |
| 16 | Vipassana | 1 | 0 | 0 |
| 17 | Fire Kasina | 0 | 0 | 1 |
| 18 | Fire Kasina | 0 | 1 | 0 |
| 19 | Fire Kasina | 1 | 0 | 0 |
| 20 | Fire Kasina | 3 | 1 | 0 |
| 21 | Fire Kasina | 0 | 0 | 1 |
| 22 | Fire Kasina | 1 | 1 | 1 |
| 23 | Fire Kasina | 4 | 0 | 0 |
| 24 | Fire Kasina | 1 | 1 | 1 |
| 25 | Fire Kasina | 0 | 2 | 2 |
| 26 | Fire Kasina | 7 | 0 | 0 |
| 27 | Fire Kasina | 0 | 3 | 1 |
| 28 | Fire Kasina | 8 | 0 | 0 |
| 29 | Fire Kasina | 6 | 1 | 0 |

Supplementary text S3

*EEG preprocessing of bad leads:*
Out of the 37 grade A cessations included in the analysis, 24 cessations contained no bad leads, 8 contained 1 bad
lead, 4 contained 2 bad leads and 1 contained 3 bad leads. Out of the 27 control segments included in the analysis, 17 segments contained no bad leads, 17 contained 1 bad lead, 1 contained 2 bad leads and 2 contained 3 bad leads. These bad Channels were not corrected for two reasons: (1) To the best of our knowledge, the effect of artifact correction algorithms such as independent component analysis (ICA) on the phase-lag index has not yet been established. As such, we conservatively chose to exclude those channels; and (2) Bad channels were discarded to keep the analysis in line with our previous EEG phase-lag index (PLI) study on meditation (Van Lutterveld et al, 2017).

Supplementary figure S4


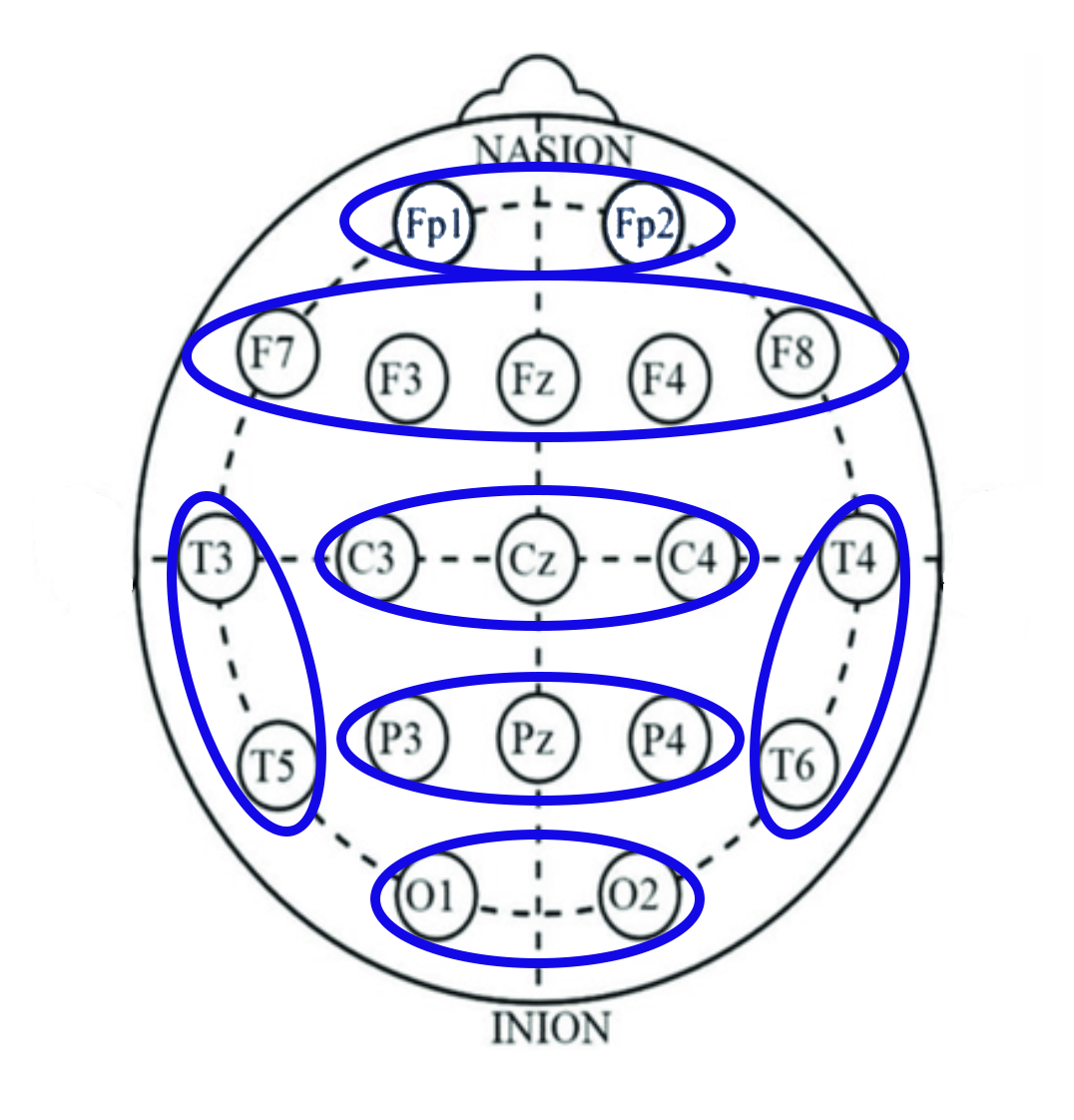


Supplementary figure S4. Sensor layout of the EEG system (Cognionics, San Diego, CA, USA) and regions-of-interest (ROIs) used in the study.

Supplementary figure and table S5.


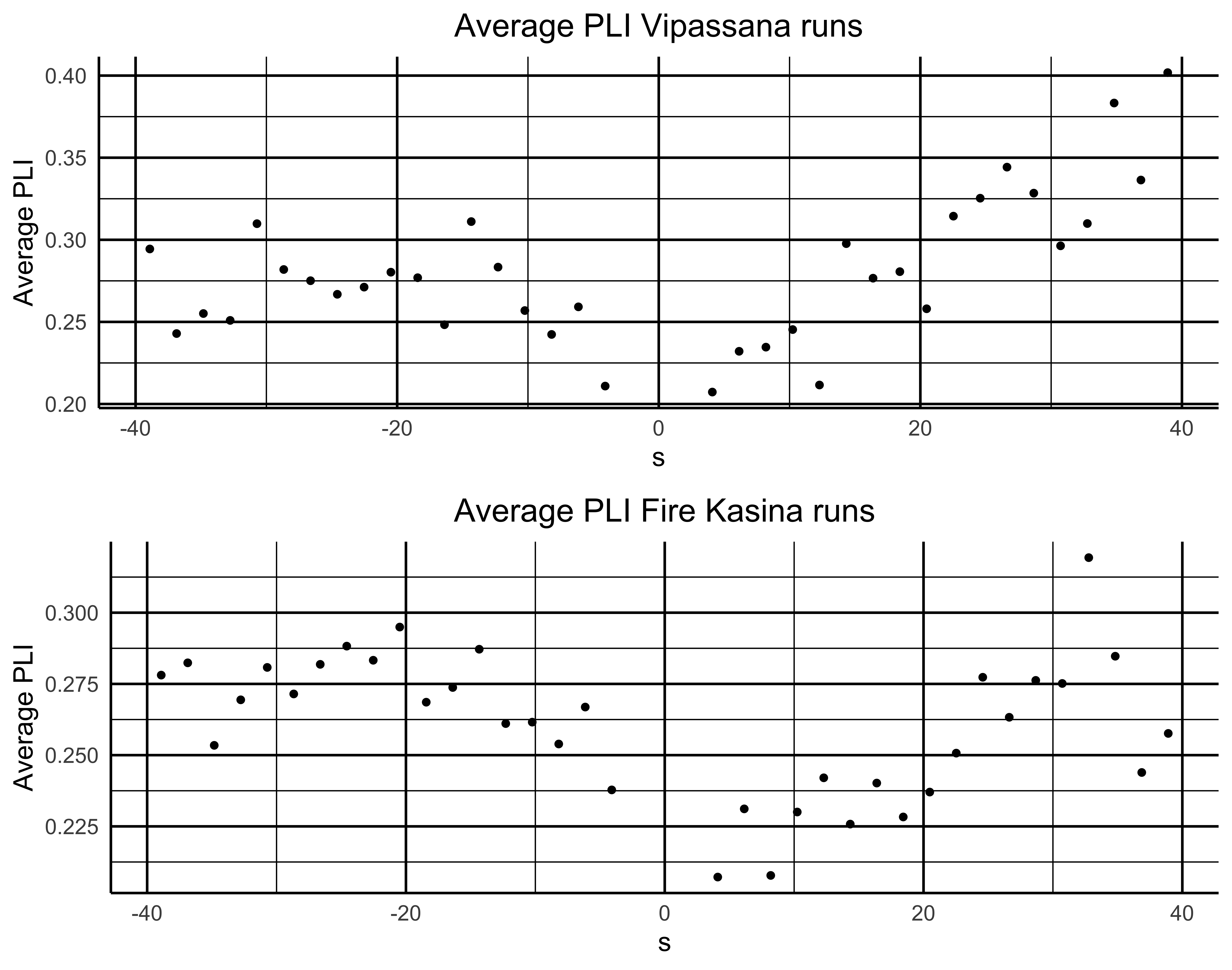


Figure S5. Functional connectivity in the alpha band for the cessation analysis for the Vipassana and fire kasina runs for the time-frames that showed significant regression findings in the main analysis. The 0 s timepoint indicates when the cessation occurred. PLI: Phase-lag index.

Table S5. Model statistics for the overall phase-lag index (PLI) regression analyses in the alpha band for the Vipassana and fire kasina runs for the time-frames that showed significant regression findings in the main analysis. CI: confidence interval.

|  | | Model statistics | | | | Parameter estimates | | | |
| --- | --- | --- | --- | --- | --- | --- | --- | --- | --- |
| Meditation type | Time frames | Degrees of freedom | *F* | *R*^2^ | *P* | B(constant) (BCa 95% CI) | *p* | B(time) (BCa 95% CI) | *p* |
| Vipassana | Pre-cessation (21.504 – 3.074 s pre-cessation, time frame 2) | 1,7 | 4.585 | 0.396 | 0.070 | 0.224 (0.186 – 0.271) | 0.004 | -0.003(-0.007 – 0.000) | 0.068 |
| Vipassana | Post-cessation (3.072 – 39.934 s post-cessation; time frames 3 and 4 combined) | 1,16 | 63.429 | 0.799 | <0.001 | 0.196 (0.172 – 0.221) | <0.001 | 0.005 (0.004 – 0.006) | <0.001 |
| Fire kasina | Pre-cessation (21.504 – 3.074 s pre-cessation, time frame 2) | 1,7 | 12.505 | 0.641 | 0.010 | 0.237 (0.223 – 0.258) | <0.001 | -0.002 (-0.004 – -0.001) | 0.033 |
| Fire kasina | Post-cessation (3.072 – 39.934 s post-cessation; time frames 3 and 4 combined) | 1,16 | 21.494 | 0.573 | <0.001 | 0.207 (0.190 – 0.221) | <0.001 | 0.002 (0.001 – 0.003) | 0.002 |

Supplementary table S6. *P < 0.05

|  | Cessations | | Control | |
| --- | --- | --- | --- | --- |
|  | -21.5 to -3.1sec | 3.1 to 39.9 sec | -21.5 to -3.1sec | 3.1 to 39.9 sec |
| Frontopolar - Frontal | 0.041* | 0.015* | 0.124 | 0.656 |
| Frontopolar – Central | 0.091 | <0.001* | 0.652 | 0.391 |
| Frontopolar – Occipital | 0.044* | <0.001* | 0.801 | 0.309 |
| Frontopolar – Parietal | 0.010* | <0.001* | 0.931 | 0.394 |
| Frontopolar – Left Temporal | 0.018* | <0.001* | 0.819 | 0.307 |
| Frontopolar – Right Temporal | 0.590 | <0.001* | 0.488 | 0.358 |
| Frontal – Central | 0.252 | <0.001* | 0.399 | 0.859 |
| Frontal – Occipital | 0.036* | <0.001* | 0.998 | 0.227 |
| Frontal – Parietal | 0.061 | <0.001* | 0.818 | 0.335 |
| Frontal – Left Temporal | 0.024* | <0.001* | 0.795 | 0.339 |
| Frontal – Right Temporal | 0.298 | <0.001* | 0.528 | 0.933 |
| Central – Occipital | 0.112 | <0.001* | 0.794 | 0.092 |
| Central – Parietal | 0.065 | <0.001* | 0.933 | 0.152 |
| Central – Left Temporal | 0.047* | 0.007* | 0.513 | 0.691 |
| Central – Right Temporal | 0.246 | 0.006* | 0.950 | 0.455 |
| Occipital – Parietal | 0.052 | <0.001* | 0.919 | 0.146 |
| Occipital – Left Temporal | 0.095 | 0.004* | 0.804 | 0.610 |
| Occipital – Right Temporal | 0.696 | <0.001* | 0.246 | 0.415 |
| Parietal – Left Temporal | 0.279 | 0.002* | 0.811 | 0.287 |
| Parietal – Right Temporal | 0.223 | 0.040* | 0.191 | 0.973 |
| Left Temporal – Right Temporal | 0.432 | 0.108 | 0.205 | 0.708 |

Supplementary references

Ingram, D. (2018) Mastering the core teachings of the Buddha, 2nd ed. London, UK: Aeon Books Ltd.
Ñāṇamoli, B. (2010). Visuddhimagga: the path of purification. Kandy: Buddhist Publication Society.

van Lutterveld, R., van Dellen, E., Pal, P., Yang, H., Stam, C. J., & Brewer, J. (2017). Meditation is associated with

increased brain network integration. NeuroImage, 158(May), 18–25. https://doi.org/10.1016/j.neuroimage.2017.06.071
